# Pharmacovigilance study of the association between dipeptidyl peptidase–4 inhibitors and angioedema using the FDA Adverse Event Reporting System (FAERS)

**DOI:** 10.1038/s41598-022-17366-x

**Published:** 2022-07-30

**Authors:** Katsuhiro Ohyama, Junichiro Shindo, Tomohiro Takahashi, Hironori Takeuchi, Yusuke Hori

**Affiliations:** 1 Center for Experiential Pharmacy Practice, School of Pharmacy, Tokyo University of Pharmacy and Life Sciences, 1432-1 Horinouchi, Hachioji, Tokyo Japan; 2 Hospital Pharmacy, Tokyo Medical University Hospital, 6-7-1, Nishishinjuku, Shinjuku-ku, Tokyo Japan

**Keywords:** Adverse effects, Drug therapy

## Abstract

Dipeptidyl peptidase-4 (DPP-4) plays a minor role in degrading vasoactive peptides that cause angioedema when angiotensin-converting enzyme (ACE) is present and fully functional. This study investigated the association between DPP-4 inhibitors (DPP-4Is) and angioedema, including cases where the concomitant use of ACE inhibitors (ACEIs) was absent. We obtained data from the US Food and Drug Administration Adverse Event Reporting System and performed a disproportionality analysis, using the reporting odds ratio (ROR) and information component (IC) for signal detection in patients aged ≥ 40 years, stratified by age group and sex. No signal was detected for DPP-4Is when the entire dataset was analyzed. However, a signal was detected for the entire female subset group, the three stratified female groups aged ≥ 60 years, and males in their 40 s. After excluding the data of concomitant ACEI users, most ROR and IC values were lower and significant only for females in their 60 s and males aged ≥ 80 years. Regarding individual DPP-4Is signals, those detected for saxagliptin and sitagliptin in some age groups disappeared after excluding the data of ACEI users. Notably, linagliptin was the only DPP-4I where signals were detected in most female groups, regardless of age and without concomitant ACEI use. Our findings suggest that some DPP-4Is were associated with a higher reporting of angioedema as per age and sex, even in the absence of concomitant ACEI use.

## Introduction

Angioedema is deep, localized edema in the subcutaneous tissues or submucosa of the airways, intestinal tract, or other organs^1, 2^. It often leads to life-threatening respiratory distress and asphyxia if the site of onset is in the pharynx, larynx, or airway^1, 3^. An increased risk of angioedema is associated with African American ethnicity, the female sex, chronic heart failure or coronary artery disease, and a history of smoking^4–6^. On the other hand, the risk of angioedema is significantly decreased in patients with diabetes mellitus^4, 6^.

Two mechanisms underlying the onset of angioedema have been proposed^7^: first, the mast cell-mediated pathway where chemical mediators (e.g., histamine and leukotriene) released from the mast cells increase the dilation and permeability of blood vessels and second, the bradykinin (BK)-mediated pathway where BK—a potent vasoactive substance—directly affects vascular permeability via its B_2_ receptor and also stimulates the release of the vasoactive peptide substance P (SP) from sensory nerves^8^. SP interacts with neurokinin 1 receptor, resulting in increased vascular permeability; this activates various signal transduction systems and is involved in a wide range of physiological functions such as neurogenic inflammation and pain.

Although there are various mechanisms that cause angioedema, one of the major causes are drugs, of which angiotensin-converting enzyme inhibitors (ACEIs)^3, 5, 9^ are the most well-known and thought to be induced via vasoactive peptides. The inhibition of angiotensin-converting enzyme (ACE; kininase II) consequently inhibits BK and SP degradation^4, 10^. Dipeptidyl peptidase-4 (DPP-4) is an enzyme that degrades peptides with a proline or alanine at the penultimate amino position^11^, such as BK and SP, and DPP-4 also degrades these peptides^11–13^. On the other hand, DPP-4 is also responsible for the degradation of incretin hormones, such as gastric inhibitory polypeptide and glucagon-like peptide-1, which stimulate insulin secretion. Thus, DPP-4 inhibitors (DPP-4Is) are widely used as antidiabetic drugs.

DPP-4 plays a minor role in the degradation of vasoactive peptides when ACE is present and fully functional. Therefore, special attention must be paid to those patients receiving concomitant treatment with DPP-4Is and ACEIs due to the high risk of developing angioedema^14–16^; this association has been the topic of many studies^4, 10–13^. However, it was proposed that the reduced DPP-4 activity itself may predispose individuals to angioedema^10^, and, in fact, angioedema as the result of DPP-4I administration without concomitant use of ACEIs has been reported^17–19^. Thus, it is unclear whether DPP-4I itself contributes to angioedema development, and few studies have examined the association of DPP-4I with angioedema.

Our study evaluated the association between DPP-4I and angioedema using data obtained from the US Food and Drug Administration (FDA) Adverse Event Reporting System (FAERS), which we stratified into groups according to age and sex. Additionally, to exclude the effect of concomitant ACEI use on the development of DPP-4I-associated angioedema, we also analyzed the data after excluding all ACEI users.

## Materials and methods

### Data source

We evaluated FAERS data from January 1, 2013 to December 31, 2019. The FAERS database consists of seven datasets: patient demographic and administrative information (DEMO), drug/biologic information (DRUG), adverse events (REAC), indications for use/diagnosis (INDI), patient outcomes, report sources, and start and end dates of drug therapy. Given that the FAERS data contains generic names and the respective brand names used in the reporting countries, all drug names in the DRUG table were changed to their respective generic names in accordance with the drug database Drugs.com (https://www.drugs.com) and its name-collecting functions. In case of redundant case identification numbers in the FAERS data, we applied the latest case identification numbers.

### Patient background

We recalculated the patients’ ages according to the age code in DEMO. We excluded all patients aged < 40 years as well as those for whom there was no information pertaining to their sex. Subsequently, the sample was divided by decade of age, 40 s to 70 s, and those older than 80 years. The main indications were determined by combining DEMO with INDI and SMQs (SMQ 20000147, SMQ 20000041, and SMQ 20000026) were used to define the indications. The reporting country of the patients was according to the description of DEMO.

### Drugs of interest

The drugs of interest comprised seven DPP-4Is that are prescribed for daily use (alogliptin, anagliptin, linagliptin, saxagliptin, sitagliptin, teneligliptin, and vildagliptin), 14 ACEIs (alacepril, benazepril, captopril, cilazapril, delapril, enalapril, fosinopril, imidapril, lisinopril, perindopril, quinapril, ramipril, temocapril, and trandolapril), including combined formulations registered in the FAERS. Because of the small number of registered reports of adverse events associated with omarigliptin and trelagliptin, they were excluded from the analysis.

### Definition of angioedema

The adverse events in REAC are coded using preferred terms (PTs) taken from the Medical Dictionary for Regulatory Activities (MedDRA) terminology. We defined the adverse event by the narrow scope of *angioedema* (coded SMQ 20000024) with 46 PTs in MedDRA v24.0.

### Data analysis

We used disproportionality analyses with different algorithms to evaluate the associations between DPP-4Is and angioedema by the reporting odds ratio (ROR) value, which is a non-Bayesian frequentist method^20^, and information component (IC) values, which is a Bayesian method^21^, to detect signals. We calculated the signal scores using a case/non-case method^22–24^.

All statistical analyses and data visualization were performed using JMP Pro v13.2.1 software (SAS Institute, Cary, NC, USA). A signal was considered as detected when the lower limit of the 95% confidence interval (CI) of the ROR was > 1 and that of the IC was > 0.

## Results

### Patient background

After deleting redundant numbers, reports with missing information on age or sex, and patients aged < 40 years from the FAERS data, a total of 3,701,618 reports remained; we used this for the analysis. The characteristics of the patient cohort are presented in Table 1. There were 83,481 reports for angioedema (SMQ 20000024).

**Table 1:** Characteristics of the patient cohort in this study.

Characteristic	Number of reports	%
Total	3,701,618	100.0
**Sex**		
Female	2,228,865	60.0
**Age**		
40 s	559,339	15.1
50 s	891,368	24.1
60 s	1,024,869	27.7
70 s	785,588	21.2
≥ 80 s	440,454	11.9
**Indication**		
Hypertension	186,457	5.0
Diabetes mellitus	215,629	5.8
Dyslipidemia	111,265	3.0
**Reporter country**		
United States	2,318,705	62.6
Unknown	193,581	5.2
Japan	159,025	4.3
France	149,108	4.0
Great Britain (United Kingdom)	142,747	3.9
Canada	121,700	3.3
Germany	92,027	2.5
Italy	83,702	2.3
Spain	39,013	1.1
Brazil	35,853	1.0

### Disproportionality analysis of the whole dataset for DPP-4I-associated angioedema

The results of the disproportionality analysis of the whole dataset revealed that DPP-4Is were not associated with angioedema (ROR: 1.05, 95% CI: 0.99–1.11; IC: 0.07, 95% CI: − 0.02 to 0.15).

### Analysis of data subsets stratified by age and sex

**Figure 1:**
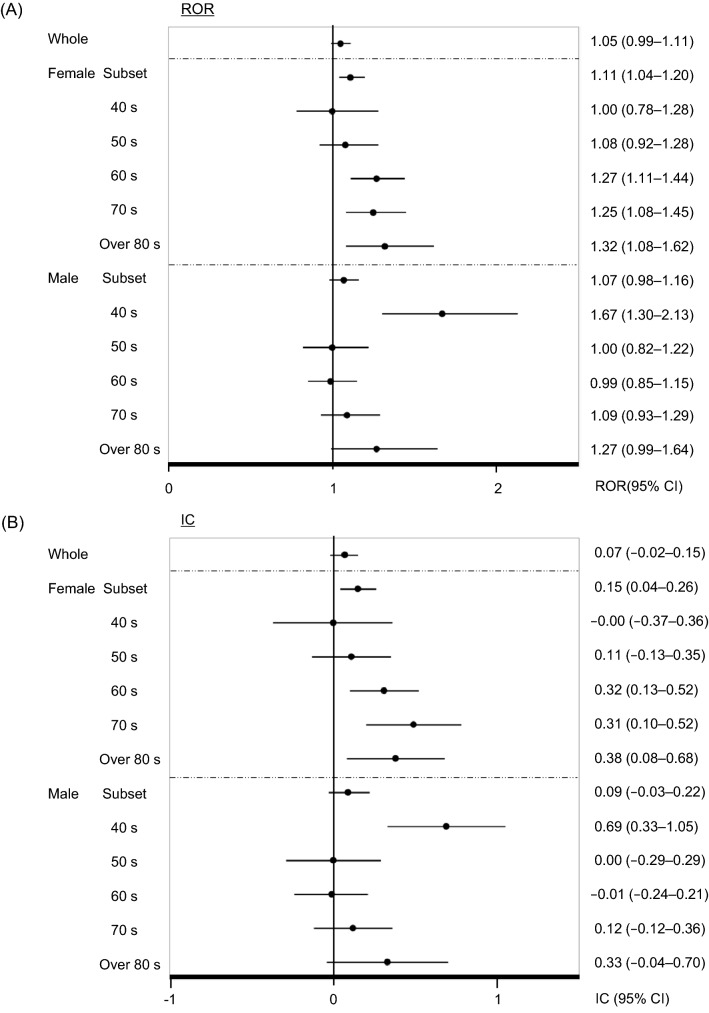
Analysis of data subsets for DPP-4I-associated angioedema stratified by age and sex. (**A**) ROR: Reporting odds ratio, (**B**) IC: Information component. CI: Confidence interval.

The results of the data analysis stratified for age and sex are presented in Fig. 1 and Table 2. The analysis for all DPP-4Is revealed signals in the whole female subset (ROR: 1.11, 95% CI: 1.04–1.20; IC: 0.15, 95% CI: 0.04–0.26); females in their 60 s (ROR: 1.27, 95% CI: 1.11–1.44; IC: 0.32, 95% CI: 0.13–0.52), 70 s (ROR: 1.25, 95% CI: 1.08–1.45; IC: 0.31, 95% CI: 0.10–0.52) and over 80 s (ROR: 1.32, 95% CI: 1.08–1.62; IC: 0.38, 95% CI: 0.08–0.68); and males in their 40 s (ROR: 1.67, 95% CI: 1.30–2.13; IC: 0.69, 95% CI: 0.33–1.05). Individually, there was an association between angioedema and the use of linagliptin for females in their 50 s (ROR: 1.67, 95% CI: 1.19–2.35; IC: 0.68, 95% CI: 0.19–1.18), 60 s (ROR: 1.94, 95% CI: 1.49–2.53; IC: 0.90, 95% CI: 0.51–1.29), 70 s (ROR: 1.77, 95% CI: 1.33–2.35; IC: 0.77, 95% CI: 0.36–1.19) and over 80 s (ROR: 2.07, 95% CI: 1.45–2.94; IC: 0.97, 95% CI: 0.46–1.97); sitagliptin for females in their 60 s (ROR: 1.21, 95% CI: 1.03–1.43; IC: 0.27, 95% CI: 0.02–0.51); and saxagliptin for females in their over 80 s (ROR: 2.62, 95% CI: 1.53–4.48; IC: 1.20, 95% CI: 0.44–1.97). On the other hand, we found an association between angioedema and males in their 40 s receiving linagliptin (ROR: 2.04, 95% CI: 1.14–3.64; IC: 0.88, 95% CI: 0.06–1.70); males in their 40 s and over 80 s, sitagliptin (ROR: 1.85, 95% CI: 1.38–2.48, IC: 0.83; 95% CI: 0.40–1.25 and ROR: 1.58, 95% CI: 1.16–2.16; IC: 0.62, 95% CI: 0.18–1.07, respectively); and males in their 50 s, saxagliptin (ROR: 2.07, 95% CI: 1.34–3.21; IC: 0.95, 95% CI: 0.32–1.58) (Table 2).

**Table 2:** Association between DPP-4 inhibitors and angioedema by stratified age and sex with whole data set.

DPP-4 inhibitor	Female				Male			
	Number of cases	Number of total users	ROR (95% CI)	IC (95% CI)	Number of cases	Number of total users	ROR (95% CI)	IC (95% CI)
**Subset**								
	731	25,946	1.11 (1.04–1.20)^a^	0.15 (0.04–0.26)^a^	555	28,497	1.07 (0.98–1.16)	0.09 (− 0.03–0.22)
**40 s**								
Subset	63	1889	1.00 (0.78–1.28)	− 0.00 (− 0.37–0.36)	67	1874	1.67 (1.30–2.13)^a^	0.69 (0.33–1.05)^a^
Alogliptin	2	55	1.09 (0.27–4.48)	0.06 (− 1.65–1.77)	1	83	0.54 (0.08–3.91)	− 0.51 (− 2.57–1.56)
Anagliptin	0	4	–	–	0	11	–	–
Linagliptin	11	281	1.18 (0.64–2.15)	0.20 (− 0.65–1.05)	12	275	2.04 (1.14–3.64)^a^	0.88 (0.06–1.70)^a^
Saxagliptin	3	198	0.44 (0.14–1.39)	− 0.94 (− 2.39–0.52)	5	191	1.20 (0.49–2.92)	0.20 (− 0.99–1.40)
Sitagliptin	47	1254	1.13 (0.84–1.51)	0.16 (− 0.26–0.59)	47	1190	1.85 (1.38–2.48)^a^	0.83 (0.40–1.25)^a^
Teneligliptin	0	19	–	–	1	27	1.72 (0.23–12.7)	0.31 (− 1.80–2.42)
Vildagliptin	1	112	0.26 (0.04–1.87)	− 1.26 (− 3.31–0.80)	2	128	0.71 (0.18–2.87)	− 0.35 (− 2.04–1.33)
**50 s**								
Subset	147	4767	1.08 (0.92–1.28)	0.11 (− 0.13–0.35)	102	5073	1.00 (0.82–1.22)	0.00 (− 0.29–0.29)
Alogliptin	6	135	1.58 (0.70–3.59)	0.52 (− 0.60–1.64)	8	206	1.97 (0.97–4.00)	0.80 (− 0.18–1.79)
Anagliptin	0	10	–	–	1	16	3.25 (0.43–24.6)	0.58 (− 1.58–2.73)
Linagliptin	35	749	1.67 (1.19–2.35)^a^	0.68 (0.19–1.18)^a^	17	846	1.00 (0.62–1.62)	0.00 (− 0.69–0.69)
Saxagliptin	7	501	0.48 (0.23–1.02)	− 0.94 (− 1.97–0.09)	21	516	2.07 (1.34–3.21)^a^	0.95 (0.32–1.58)^a^
Sitagliptin	91	3082	1.04 (0.84–1.28)	0.05 (− 0.26–0.35)	50	3157	0.78 (0.59–1.04)	− 0.34 (− 0.75–0.07)
Teneligliptin	0	20	–	–	1	59	0.84 (0.12–6.08)	− 0.14 (− 2.21–1.93)
Vildagliptin	8	320	0.87 (0.43–1.76)	− 0.18 (− 1.15–0.80)	4	368	0.54 (0.20–1.44)	− 0.75 (− 2.05–0.55)
**60 s**								
Subset	234	7688	1.27 (1.11–1.44)^a^	0.32 (0.13–0.52)^a^	172	8927	0.99 (0.85–1.15)	− 0.01 (− 0.24–0.21)
Alogliptin	8	244	1.36 (0.67–2.76)	0.37 (− 0.61–1.35)	4	372	0.55 (0.20–1.47)	− 0.72 (− 2.02–0.58)
Anagliptin	0	13	–	–	2	26	4.20 (0.99–17.8)	0.98 (− 0.78–2.73)
Linagliptin	57	1241	1.94 (1.49–2.53)^a^	0.90 (0.51–1.29)^a^	35	1496	1.21 (0.86–1.69)	0.26 (− 0.23–0.74)
Saxagliptin	16	736	0.89 (0.54–1.47)	− 0.15 (− 0.86–0.56)	15	835	0.92 (0.55–1.54)	− 0.11 (− 0.84–0.62)
Sitagliptin	142	4856	1.21 (1.03–1.43)^a^	0.27 (0.02–0.51)^a^	104	5423	0.99 (0.81–1.20)	− 0.02 (− 0.31–0.26)
Teneligliptin	0	54	–	–	1	124	0.41 (0.06–2.93)	− 0.78 (− 2.84–1.28)
Vildagliptin	16	666	0.99 (0.60–1.63)	− 0.02 (− 0.73–0.69)	12	797	0.77 (0.44–1.36)	− 0.35 (− 1.15–0.46)
**70 s**								
Subset	191	7229	1.25 (1.08–1.45)^a^	0.31 (0.10–0.52)^a^	151	8549	1.09 (0.93–1.29)	0.12 (− 0.12–0.36)
Alogliptin	8	305	1.24 (0.61–2.50)	0.26 (− 0.72–1.24)	8	451	1.10 (0.54–2.21)	0.11 (− 0.86–1.08)
Anagliptin	0	11	–	–	0	19	–	–
Linagliptin	49	1326	1.77 (1.33–2.35)^a^	0.77 (0.36–1.19)^a^	35	1606	1.36 (0.97–1.90)	0.41 (− 0.07–0.90)
Saxagliptin	12	580	0.97 (0.55–1.72)	− 0.04 (− 0.85–0.77)	14	623	1.40 (0.82–2.38)	0.43 (− 0.32–1.19)
Sitagliptin	108	4355	1.17 (0.97–1.42)	0.22 (− 0.06–0.50)	83	4884	1.05 (0.84–1.31)	0.07 (− 0.25–0.39)
Teneligliptin	2	99	0.95 (0.23–3.84)	− 0.06 (− 1.75–1.63)	0	161	–	–
Vildagliptin	19	666	1.35 (0.86–2.13)	0.40 (− 0.26–1.05)	14	934	0.92 (0.54–1.57)	− 0.11 (− 0.86–0.65)
**Over 80 s**								
Subset	96	4373	1.32 (1.08–1.62)^a^	0.38 (0.08–0.68)^a^	63	4074	1.27 (0.99–1.64)	0.33 (− 0.04–0.70)
Alogliptin	1	227	0.26 (0.04–1.85)	− 1.27 (− 3.32–0.78)	2	235	0.69 (0.17–2.78)	− 0.38 (− 2.06–1.30)
Anagliptin	0	7	–	–	0	16	–	–
Linagliptin	32	944	2.07 (1.45–2.94)^a^	0.97 (0.46–1.48)^a^	13	857	1.24 (0.72–2.15)	0.28 (− 0.50–1.06)
Saxagliptin	14	328	2.62 (1.53–4.48)^a^	1.20 (0.44–1.97)^a^	1	214	0.38 (0.05–2.69)	− 0.86 (− 2.91–1.19)
Sitagliptin	42	2366	1.06 (0.78–1.44)	0.08 (− 0.37–0.53)	42	2190	1.58 (1.16–2.16)^a^	0.62 (0.18–1.07)^a^
Teneligliptin	0	61	–	–	0	63	–	–
Vildagliptin	9	517	1.04 (0.54–2.01)	0.05 (− 0.88–0.97)	7	558	1.02 (0.48–2.16)	0.02 (− 1.00–1.05)

*DPP-4* dipeptidyl peptidase-4, *ROR* reporting odds ratio, *IC* information component, *CI* confidence interval.

^a^Signal was detected.

### Analysis in subset data stratified by age and sex for patients without concomitant ACEI treatment

**Figure 2:**
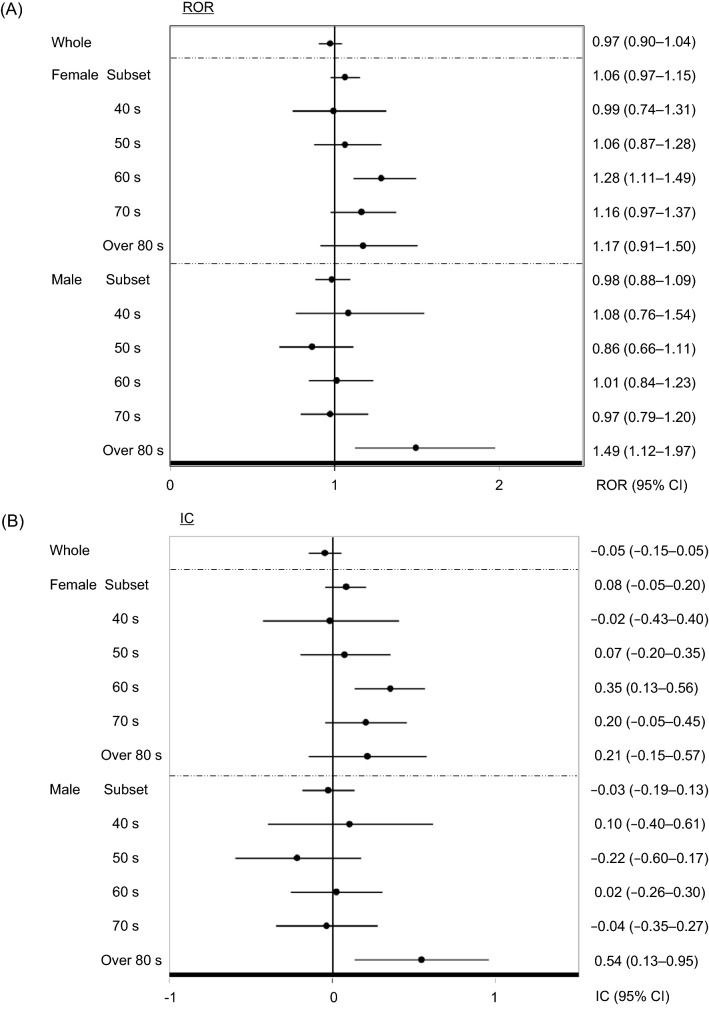
Analysis in subset data stratified by age and sex for patients without concomitant ACEI treatment. (**A**) ROR: Reporting odds ratio, (**B**) IC: Information component. CI: Confidence interval.

Overall, 229,376 of the 3,701,618 (6.2%) records of patients using DPP-4Is showed that they received concomitant treatment with ACEIs. The remaining 3,472,242 patients usedDPP-4Is alone; of these 72,369 had adverse event reports related to angioedema. Our analysis of the association between DPP-4I and angioedema revealed that most ROR and IC values (95% CI) were less than those obtained when the whole dataset was analyzed (Fig. 2 and Table 3). In the analysis for all DPP-4Is, a significant association was found only for females in their 60 s (ROR: 1.28, 95% CI: 1.11–1.49; IC: 0.35, 95% CI: 0.13–0.56) and males over 80 s (ROR: 1.49, 95% CI: 1.12–1.97; IC: 0.54, 95% CI: 0.13–0.95) (Fig. 2). Individually, there was an association between angioedema and the use of linagliptin for females in their 50 s (ROR: 1.59, 95% CI: 1.08–2.36; IC: 0.62, 95% CI: 0.05–1.18), 60 s (ROR: 2.12, 95% CI: 1.58–2.83; IC: 1.01, 95% CI: 0.59–1.43), 70 s (ROR: 1.62, 95% CI: 1.15–2.27, IC: 0.65, 95% CI: 0.15–1.14), and over 80 s (ROR: 2.13, 95% CI: 1.44–3.15; IC: 1.00, 95% CI: 0.43–1.57). On the other hand, we found an association between angioedema and the use of linagliptin in males in their 40 s (ROR: 2.30, 95% CI: 1.22–4.34; IC: 1.00 95% CI: 0.11–1.90) and 70 s (ROR: 1.53, 95% CI: 1.04–2.26; IC: 0.58 95%, CI: 0.01–1.14) as well as the use of sitagliptin for males in their over 80 s (ROR: 1.96, 95% CI: 1.39–2.27; IC: 0.91 95% CI: 0.41–1.40) (Table 3).

**Table 3:** Association between DPP-4 inhibitors without concomitant ACE inhibitor use and angioedema by stratified age and sex.

DPP-4 inhibitor	Female				Male			
	Number of cases	Number of total users	ROR (95% CI)	IC (95% CI)	Number of cases	Number of total users	ROR (95% CI)	IC (95% CI)
**Subset**								
	532	20,928	1.06 (0.97–1.15)	0.08 (− 0.05–0.20)	335	22,267	0.98 (0.88–1.09)	− 0.03 (− 0.19–0.13)
**40 s**								
Subset	49	1511	0.99 (0.74–1.31)	− 0.02 (− 0.43–0.40)	32	1468	1.08 (0.76–1.54)	0.10 (− 0.40–0.61)
Alogliptin	2	51	1.20 (0.29–4.95)	0.15 (− 1.56–1.86)	1	79	0.62 (0.09–4.47)	− 0.39 (− 2.45–1.68)
Anagliptin	0	4	–	–	0	11	–	–
Linagliptin	9	236	1.17 (0.60–2.28)	0.19 (− 0.74–1.12)	10	221	2.30 (1.22–4.34)^a^	1.00 (0.11–1.90)^a^
Saxagliptin	2	142	0.42 (0.10–1.70)	− 0.92 (− 2.61–0.76)	4	140	1.43 (0.53–3.86)	0.38 (− 0.94–1.69)
Sitagliptin	36	989	1.11 (0.80–1.55)	0.14 (− 0.34–0.63)	15	909	0.81 (0.49–1.36)	− 0.28 (− 1.01–0.45)
Teneligliptin	0	19	–	–	1	27	1.87 (0.25–13.8)	0.35 (− 1.76–2.47)
Vildagliptin	0	96	–	–	2	107	0.92 (0.23–3.74)	− 0.09 (− 1.77–1.60)
**50 s**								
Subset	110	3773	1.06 (0.87–1.28)	0.07 (− 0.20–0.35)	57	3860	0.86 (0.66–1.11)	− 0.22 (− 0.60–0.17)
Alogliptin	6	108	2.07 (0.91–4.71)	0.80 (− 0.32–1.93)	6	181	1.96 (0.87–4.43)	0.76 (− 0.35–1.87)
Anagliptin	0	10	–	–	1	14	4.40 (0.58–33.6)	0.67 (− 1.50–2.84)
Linagliptin	26	600	1.59 (1.08–2.36)^a^	0.62 (0.05–1.18)^a^	8	662	0.70 (0.35–1.40)	− 0.46 (− 1.43–0.51)
Saxagliptin	6	378	0.57 (0.25–1.27)	− 0.71 (− 1.82–0.39)	9	358	1.48 (0.76–2.86)	0.48 (− 0.45–1.41)
Sitagliptin	66	2431	0.98 (0.77–1.25)	− 0.03 (− 0.39–0.33)	28	2348	0.69 (0.47–1.00)	− 0.51 (− 1.05–0.03)
Teneligliptin	0	18	–	–	1	58	1.00 (0.14–7.25)	− 0.01 (− 2.09–2.06)
Vildagliptin	6	263	0.82 (0.37–1.84)	− 0.25 (− 1.35–0.86)	4	312	0.74 (0.28–1.99)	− 0.35 (− 1.65–0.95)
**60 s**								
Subset	180	6116	1.28 (1.11–1.49)^a^	0.35 (0.13–0.56)^a^	107	6819	1.01 (0.84–1.23)	0.02 (− 0.26–0.30)
Alogliptin	5	217	1.00 (0.41–2.42)	− 0.01 (− 1.20–1.18)	5	336	0.96 (0.40–2.32)	− 0.05 (− 1.24–1.14)
Anagliptin	0	12	–	–	1	25	2.65 (0.36–19.6)	0.51 (− 1.61–2.63)
Linagliptin	48	1008	2.12 (1.58–2.83)^a^	1.01 (0.59–1.43)^a^	23	1151	1.30 (0.86–1.96)	0.35 (− 0.25–0.95)
Saxagliptin	14	570	1.06 (0.63–1.81)	0.08 (− 0.68–0.83)	8	609	0.85 (0.42–1.70)	− 0.21 (− 1.18–0.75)
Sitagliptin	100	3774	1.15 (0.94–1.41)	0.19 (− 0.10–0.49)	63	4026	1.01 (0.79–1.30)	0.01 (− 0.35–0.38)
Teneligliptin	0	47	–	–	1	118	0.54 (0.08–3.89)	− 0.51 (− 2.56–1.55)
Vildagliptin	15	583	1.12 (0.67–1.86)	0.14 (− 0.59–0.87)	9	666	0.87 (0.45–1.68)	− 0.18 (− 1.10–0.74)
**70 s**								
Subset	134	5882	1.16 (0.97–1.37)	0.20 (− 0.05–0.45)	88	6802	0.97 (0.79–1.20)	− 0.04 (− 0.35–0.27)
Alogliptin	6	287	1.06 (0.47–2.37)	0.06 (− 1.04–1.17)	4	398	0.75 (0.28–2.02)	− 0.33 (− 1.63–0.97)
Anagliptin	0	11	–	–	0	18	–	–
Linagliptin	34	1078	1.62 (1.15–2.27)^a^	0.65 (0.15–1.14)^a^	26	1288	1.53 (1.04–2.26)^a^	0.58 (0.01–1.14)^a^
Saxagliptin	8	468	0.86 (0.43–1.73)	− 0.19 (− 1.16–0.78)	10	474	1.60 (0.86–3.00)	0.59 (− 0.29–1.47)
Sitagliptin	77	3469	1.13 (0.90–1.41)	0.16 (− 0.17–0.49)	44	3801	0.87 (0.64–1.17)	− 0.19 (− 0.63–0.24)
Teneligliptin	2	94	1.08 (0.27–4.37)	0.06 (− 1.63–1.75)	0	151	–	–
Vildagliptin	8	567	0.71 (0.35–1.42)	− 0.44 (− 1.41–0.53)	6	779	0.58 (0.26–1.29)	− 0.70 (− 1.80–0.40)
**Over 80 s**								
Subset	64	3646	1.17 (0.91–1.50)	0.21 (− 0.15–0.57)	51	3318	1.49 (1.12–1.97)^a^	0.54 (0.13–0.95)^a^
Alogliptin	1	208	0.31 (0.04–2.24)	− 1.06 (− 3.11–0.99)	2	230	0.83 (0.21–3.34)	− 0.19 (− 1.87–1.49)
Anagliptin	0	6	–	–	0	15	–	–
Linagliptin	26	825	2.13 (1.44–3.15)^a^	1.00 (0.43–1.57)^a^	12	724	1.60 (0.90–2.83)	0.60 (− 0.21–1.41)
Saxagliptin	5	242	1.37 (0.57–3.33)	0.36 (− 0.83–1.55)	1	182	0.52 (0.07–3.73)	− 0.55 (− 2.60–1.51)
Sitagliptin	30	1952	1.02 (0.71–1.46)	0.02 (− 0.50–0.55)	34	1686	1.96 (1.39–2.77)^a^	0.91 (0.41–1.40)^a^
Teneligliptin	0	57	–	–	0	57	–	–
Vildagliptin	2	427	0.31 (0.08–1.23)	− 1.32 (− 2.99–0.36)	4	480	0.79 (0.30–2.12)	− 0.27 (− 1.57–1.03)

*DPP-4* dipeptidyl peptidase-4, *ACE* angiotensin converting enzyme, *ROR* reporting odds ratio, *IC* information component, *CI* confidence interval.

^a^Signal was detected.

## Discussion

Vasoactive peptide-induced angioedema may occur not only in ACEIs but also in DPP-4Is. This study evaluated the association between DPP-4Is and angioedema, including cases with and without the concomitant use of ACEIs. When we stratified the data according to age group and sex, we detected a signal for the female subset, three female age groups with patients aged ≥ 60 s, and the group of males in their 40 s. ON excluding ACEI users from the whole dataset, we only detected a signal for females in their 60 s and males aged ≥ 80 years. Individually, signals for saxagliptin and sitagliptin, which were detected when the whole dataset was analyzed, disappeared when we excluded ACEI users. Notably, linagliptin was the only DPP-4I where a signal was detected in females regardless of age and concomitant ACEI use.

From the stratified analysis in our study, the number of detected signals for DPP-4I–associated angioedema was more in the female groups than in the male groups (Fig. 1, Table 2) and more in the elderly groups than in the middle-adulthood groups (Fig. 1, Table 2). Generally, the incidence of drug-induced angioedema has been reported as higher in females^25, 26^, and older age has been associated with a higher incidence of angioedema^27^, which supports our results.

A previous study using pharmacovigilance databases also reported the association between DPP-4I and angioedema^28, 29^. Lepelley et al. evaluated the association of an increased angioedema reporting risk using the World Health Organization’s pharmacovigilance database, reporting that exposure to DPP-4I alone was not associated with a disproportionality signal for angioedema^28^. Moreover, in another study using the Japanese Adverse Drug Event Report database, the authors also performed a disproportionality analysis to evaluate DPP-4I/ACEI-induced angioedema and concluded that DPP-4I tended to have different effects on the onset of angioedema from ACEI in clinical practice, because an inverse association of DPP-4I with angioedema was found. The difference in the conclusion could be attributed to the use of different pharmacovigilance databases, which include information on different races. Additionally, although the adverse event (angioedema) was defined according to MedDRA in both cases, there was a slight difference in the selection of PTs. Moreover, our study classified the data in more detail by stratification according to age and sex.

Some case reports have suggested that linagliptin can cause acute renal failure with hypotension and hyperkalemia when added to the treatment regimens of patients already receiving ACEIs^30, 31^. Moreover, a recent in silico and in vivo study reported that many DPP-4Is, including linagliptin, could potentially inhibit ACE in concentrations close to those required for DPP-4 inhibition^32^. These results suggest that linagliptin can inhibit two enzymes, both of which contribute to BK and SP degradation, and it may be reasonable to assume that linagliptin causes angioedema.

Conversely, it was also reported that sitagliptin inhibits both DPP-4 and ACE^32^, despite the detection of a limited signal for sitagliptin in our study (Tables 2 and 3). A possible explanation may be the different pharmacokinetics of these drugs. In other words, since the more complete or sustained ACE inhibition seen with the longer-acting agents may be detrimental^33^, DPP-4Is are suggested to inhibit ACEs for a longer-acting duration and also be more likely to cause angioedema than shorter-acting agents. The half-life of linagliptin and sitagliptin is 104–113 h^34^ and 9–14 h^35^, respectively. Further studies are necessary to determine whether such inhibitory effects occur in clinical settings.

It has been proposed that patients with a history of ACEI-induced angioedema are at risk for recurrent angioedema with DPP-4Is^36^. This may be explained by reports that the enzymatic activity of DPP-4 is innately reduced in the sera of such patients compared with the sera of ACEI-treated patients without angioedema^37–39^. Furthermore, the concentrations of enzymes involved in BK and SP degradation were reported to have drastically reduced compared with the reference range for at least a year after inhibition by DPP-4Is and ACEIs^37, 40^. Therefore, the recurrence of angioedema in patients with such a history warrants additional care, given the possible inhibition of BK and SP degradation, even without DPP-4Is and ACEIs administration.

This study suggests that the use of DPP-4Is, even in the absence of concomitant ACEI use, is associated with angioedema in clinical practice. To determine whether this is a class effect of DPP-4I, further studies are essential. In any case, clinicians should be aware of the possible association as seen in this study.

Although the analysis of a spontaneous report is a valuable method for identifying signals, there are potential limitations of such databases that may have affected the interpretation of our findings. First, adverse events are not always induced by medication. Second, angioedema is influenced by many confounding factors, such as comorbidities including chronic heart failure or coronary artery disease^4, 5^; the concomitant administration of drugs other than ACEIs, such as angiotensin receptor blockers, antibiotics, and nonsteroidal anti-inflammatory drugs^41^; and smoking^5, 6^, which we did not take into consideration in this study. Although the incidence of angioedema has been reported to be lower in patients with diabetes mellitus^4, 6^, we had to include such patients because DPP-4Is are used to treat diabetes mellitus in clinical practice. Third, as mentioned above, duplicate reports for the same case could be included in spontaneous reporting systems, and, conversely, underreporting may occur. Fourth, data are missing and drug names are frequently misspelled in the FAERS database. Additionally, concerning data mining techniques, given that control populations are not included in spontaneous reporting systems, disproportionality-based signals indicate an increased risk of adverse event reporting, not the risk of the adverse events.

## Conclusions

Although dependent on age and sex, our study findings suggest that some DPP-4Is were associated with a higher reporting of angioedema, even in the absence of the concomitant use of ACEIs. In particular, linagliptin was the only DPP-4I associated with angioedema regardless of age and the absence of concomitant ACEI use, in females. Further studies are essential to determine whether this is a class effect of DPP-4I. Our study supported the hypothesis that DPP4-Is may induce angioedema in susceptible patients, even in the absence of concomitant ACEI.

## Data Availability

The authors do not own the data because the FDA does not permit direct sharing of data from the respective adverse event reporting database. The FAERS data can be accessed directly at the following URLs: https://fis.fda.gov/extensions/FPD-QDE-FAERS/FPD-QDE-FAERS.html.

## References

[1] Byrd JB, Adam A, Brown NJ (2006). Angiotensin-converting enzyme inhibitor-associated angioedema. Immunol. Allergy Clin. North Am..

[2] Kaplan AP, Greaves MW (2005). Angioedema. J. Am. Acad. Dermatol..

[3] Bas M (2017). The angiotensin-converting-enzyme-induced angioedema. Immunol. Allergy Clin. North Am..

[4] Miller DR, Oliveria SA, Berlowitz DR, Fincke BG, Stang P, Lillienfeld DE (2008). Angioedema incidence in US veterans initiating angiotensin-converting enzyme inhibitors. Hypertension.

[5] Morimoto T, Gandhi TK, Fiskio JM, Seger AC, So JW, Cook EF, Fukui T, Bates DW (2004). An evaluation of risk factors for adverse drug events associated with angiotensin-converting enzyme inhibitors. J. Eval. Clin. Pract..

[6] Kostis JB, Kim HJ, Rusnak J, Casale T, Kaplan A, Corren J, Levy E (2005). Incidence and characteristics of angioedema associated with enalapril. Arch. Intern. Med..

[7] Caballero T, Baeza ML, Cabañas R, Campos A, Cimbollek S, Gómez-Traseira C, González-Quevedo T, Guilarte M, Jurado-Palomo GJ, Larco JI, López-Serrano MC, López-Trascasa M, Marcos C, Muñoz-Caro JM, Pedrosa M, Prior N, Rubio M, Sala-Cunill A (2011). Consensus statement on the diagnosis, management, and treatment of angioedema mediated by bradykinin. Part I. Classification, epidemiology, pathophysiology, genetics, clinical symptoms, and diagnosis. J. Investig. Allergol. Clin. Immunol..

[8] Kopp UC, Farley DM, Smith LA (1997). Bradykinin-mediated activation of renal sensory neurons due to prostaglandin-dependent release of substance P. Am. J. Physiol..

[9] Banerji A, Clark S, Blanda M, LoVecchio F, Synder B, Camargo CA (2008). Multicenter study of patients with angiotensin-converting enzyme inhibitor-induced angioedema who present to the emergency department. Ann. Allerg. Asthma Immunol..

[10] Byrd JB, Touzin K, Sile S, Gainer JV, Yu C, Nadeau J, Adam A, Brown NJ (2008). Dipeptidyl peptidase IV in angiotensin-converting enzyme inhibitor associated angioedema. Hypertension.

[11] Lambeir AM, Durinx C, Scharpé S, De Meester I (2003). Dipeptidyl-peptidase IV from bench to bedside: An update on structural properties, functions, and clinical aspects of the enzyme DPP IV. Crit. Rev. Clin. Lab Sci..

[12] Moreau ME, Garbacki N, Molinaro G, Brown NJ, Marceau F, Adam A (2005). The kallikrein-kinin system: Current and future pharmacological targets. J. Pharmacol. Sci..

[13] Russell JS, Chi H, Lantry LE, Stephens RE, Ward PE (1996). Substance P and neurokinin A metabolism by cultured human skeletal muscle myocytes and fibroblasts. Peptides.

[14] Vasekar M, Craig TJ (2012). ACE inhibitor-induced angioedema. Curr Allergy Asthma Rep.

[15] Brown NJ, Byiers S, Carr D, Maldonado M, Warner BA (2009). Dipeptidyl peptidase-IV inhibitor use associated with increased risk of ACE inhibitor associated angioedema. Hypertension.

[16] Hahn J, Trainotti S, Hoffmann TK, Greve J (2017). Drug-induced inhibition of angiotensin converting enzyme and dipeptidyl peptidase 4 results in nearly therapy resistant bradykinin induced angioedema: A case report. Am. J. Case Rep..

[17] Campo P, Fernandez TD, Canto G, Mayorga C (2013). Angioedema induced by angiotensin converting enzyme inhibitors. Curr. Opin. Allergy Clin. Immunol..

[18] Gosmanov AR, Fontenot EC (2012). Sitagliptin-associated angioedema. Diabetes Care.

[19] Saisho Y, Itoh H (2013). Dipeptidyl peptidase-4 inhibitors and angioedema: A class effect?. Diabet Med..

[20] van Puijenbroek EP, Bate A, Leufkens HG, Lindquist M, Orre R, Egberts AC (2002). A comparison of measures of disproportionality for signal detection in spontaneous reporting systems for adverse drug reactions. Pharmacoepidemiol. Drug Saf..

[21] Bate A, Lindquist M, Edwards IR, Olsson S, Orre R, Lansner A, De Freitas RM (1998). A Bayesian neural network method for adverse drug reaction signal generation. Eur. J. Clin. Pharmacol..

[22] Takada M, Fujimoto M, Hosomi K (2016). Association between benzodiazepine use and dementia: Data mining of different medical databases. Int. J. Med. Sci..

[23] Yokoyama S, Ieda S, Nagano M, Nakagawa C, Iwase M, Hosomi K, Takada M (2020). Association between oral anticoagulants and osteoporosis: Real-world data mining using a multi-methodological approach. Int. J. Med. Sci..

[24] Ohyama K, Tanaka H, Shindo J, Shibayama M, Iwata M, Hori H (2022). Association of gynecomastia with antidiabetic medications in older adults: Data mining from different national pharmacovigilance databases. Int. J. Clin. Pharmacol. Ther..

[25] Poulos LM, Waters AM, Correll PK, Loblay RH, Marks GB (2007). Trends in hospitalizations for anaphylaxis, angioedema, and urticaria in Australia, 1993–1994 to 2004–2005. J. Allergy Clin. Immunol..

[26] Malbrán E, Fernández Romero D, Juri MC, Larrauri BJ, Malbrán A (2015). Epidemiology of angioedema without wheals in an allergy and immunology center. Medicina (B Aires).

[27] Loftus PA, Tan M, Patel G, Lin J, Helman S, Badhey A, Du E, Smith RV, Fried MP, Ow TJ (2014). Risk factors associated with severe and recurrent angioedema: An epidemic linked to ACE-inhibitors. Laryngoscope.

[28] Lepelley M, Khouri C, Lacroix C, Bouillet L (2020). Angiotensin-converting enzyme and dipeptidyl peptidase-4 inhibitor-induced angioedema: A disproportionality analysis of the WHO pharmacovigilance database. J. Allergy Clin. Immunol. Pract..

[29] Noguchi Y, Murayama A, Esaki H, Sugioka M, Koyama A, Tachi T, Teramachi H (2021). Angioedema caused by drugs that prevent the degradation of vasoactive peptides: A pharmacovigilance database study. J. Clin. Med..

[30] Kutoh E (2012). Potential linagliptin-induced renal impairment. J. Med. Cases.

[31] Nandikanti DK, Gosmanova EO, Gosmanov AR (2016). Acute kidney injury associated with linagliptin. Case Rep. Endocrinol..

[32] Abouelkheir M, El-Metwally TH (2019). Dipeptidyl peptidase-4 inhibitors can inhibit angiotensin converting enzyme. Eur. J. Pharmacol..

[33] Mason NA (1990). Angiotensin-converting enzyme inhibitors and renal function. DICP Ann. Pharmacother..

[34] Sarashina A, Sesoko S, Nakashima M, Hayashi N, Taniguchi A, Horie Y, Graefe-Mody EU, Woerle HJ, Dugi KA (2010). Linagliptin, a dipeptidyl peptidase-4 inhibitor in development for the treatment of type 2 diabetes mellitus: A Phase I, randomized, double-blind, placebo-controlled trial of single and multiple escalating doses in healthy adult male Japanese subjects. Clin. Ther..

[35] Herman GA, Mistry GC, Yi B, Bergman AJ, Wang AQ, Zeng W, Chen L, Snyder K, Ruckle JL, Larson PJ, Davies MJ, Langdon RB, Gottesdiener KM, Wagner JA (2011). Evaluation of pharmacokinetic parameters and dipeptidyl peptidase-4 inhibition following single doses of sitagliptin in healthy, young Japanese males. Br. J. Clin. Pharmacol..

[36] Hermanrud T, Bygum A, Rasmussen ER (2017). Recurrent angioedema associated with pharmacological inhibition of dipeptidyl peptidase IV. BMJ Case Rep..

[37] Beaudouin E, Defendi F, Picaud J, Drouet C, Ponard D, Moneret-Vautrin DA (2020). Iatrogenic angioedema associated with ACEi, sitagliptin, and deficiency of 3 enzymes catabolizing bradykinin. Eur. Ann. Allergy Clin. Immunol..

[38] Nussberger J, Cugno M, Cicardi M (2002). Bradykinin-mediated angioedema. N. Engl. J. Med..

[39] Byrd JB, Adam A, Brown NJ (2020). Angiotensin-converting enzyme inhibitor-associated angioedema. Immunol. Allergy Clin. North Am..

[40] Kaplan AP (2008). Angioedema. World Allergy Organ. J..

[41] Stone C, Brown NJ (2017). Angiotensin-converting enzyme inhibitor and other drug-associated angioedema. Immunol. Allergy Clin. North Am..

